# Imatinib Mesylate Effectiveness in Chronic Myeloid Leukemia with Additional Cytogenetic Abnormalities at Diagnosis among Black Africans

**DOI:** 10.1155/2013/901589

**Published:** 2013-05-29

**Authors:** Tolo Diebkilé Aïssata, Duni Sawadogo, Clotaire Nanho, Boidy Kouakou, N'dogomo Meité, N'Dhatz Emeuraude, Ayémou Roméo, Sekongo Yassongui Mamadou, Paul Kouéhion, Konan Mozart, Gustave Koffi, Ibrahima Sanogo

**Affiliations:** ^1^Department of Clinical Hematology, Yopougon Teaching Hospital, P.O. Box 632, Abidjan 21, Cote d'Ivoire; ^2^Department of Biological Hematology, Yopougon Teaching Hospital, P.O. Box 632, Abidjan 21, Cote d'Ivoire

## Abstract

Imatinib mesylate provides good results in the treatment of CML in general. But what about the results of this treatment in CML associated with additional cytogenetic abnormalities at diagnosis among black Africans? For this, we retrospectively studied 27 cases of CML associated with additional cytogenetic abnormalities, diagnosed in the department of clinical hematology of the University Hospital of Yopougon in Côte d'Ivoire, from May 2005 to October 2011. The age of patients ranged from 13 to 68 years, with a mean age of 38 years and a sex ratio of 2. Patients were severely symptomatic with a high Sokal score of 67%. CML in chronic phase accounted for 67%. The prevalence of additional cytogenetic abnormalities was 29.7%. There were variants of the Philadelphia chromosome (18.5%), trisomy 8 (14.8%), complex cytogenetic abnormalities (18.5%), second Philadelphia chromosome (14.8%), and minor cytogenetic abnormalities (44.4%). Complete hematologic remission was achieved in 59%, with 52% of major cytogenetic remission. The outcome was fatal in 37% of patients. Death was related in 40% to hematologic toxicity and in 30% to acutisation. The median survival was 40 months.

## 1. Introduction

Chronic myeloid leukemia (CML) is characterized by the predominant proliferation of cells of grainy line and by the existence of a cytogenetic abnormality that is the translocation t (9; 22) (q34; q11) with BCR/ABL rearrangement.

If CML develops inexorably towards the acute transformation, the time necessary for this transformation is variable. Twenty five percent of patients will survive more than 5 years, 5% over 10 years. For others, the acute transformation occurs immediately or very soon after the diagnosis of the disease.

This difference in overall survival may be partly explained by the Sokal score but also by the existence or not of additional cytogenetic abnormalities at diagnosis. Major additional cytogenetic abnormalities at diagnosis have a negative impact on survival and mean progression to the accelerated or blast phase [1–4].

Currently, the first-line treatment of this CML is based on imatinib mesylate. Imatinib mesylate or STI-571 or Glivec was discovered right in 1988 as a highly potent and selective inhibitor of ABL tyrosine kinases. This drug has become the first-line treatment of Ivorian patients with CML and with low income, since GIPAP (Glivec International Patient Assistance Program) kindly provides Côte d'Ivoire with it. Some resistance to this treatment may be associated with additional cytogenetic abnormalities [1, 5].

The aim of our study was to determine the effectiveness of imatinib mesylate in the CML with additional cytogenetic abnormalities. The study was approved by an ethics committee and was carried out in accordance with the Declaration of Helsinki. A written consent was signed before inclusion in the protocol.

## 2. Patients and Methods 

Our study was carried out in the department of clinical hematology of the University Hospital of Yopougon in Abidjan, Côte d'Ivoire. It was retrospective and descriptive. It involved records of in-patients or out-patients from May 2005 to October 2011. All in-patients or out-patients with CML diagnosed by blood count, myelogram, and cytogenetic or molecular biology with additional chromosomal abnormality and treated by imatinib mesylate were included. Twenty-seven CML patients with additional cytogenetic abnormalities treated by imatinib mesylate were retained.

Each medical record was exploited through a personal survey form with collection of epidemiological data (age, sex), clinical data (lymphadenopathy, splenomegaly, hepatomegaly, performance status, bone pain, and fever), biological data (white blood cell count, platelet count, hemoglobin count, blood blasts rates, blood promyelocytes rates, and the result of cytogenetic or molecular biology), evolutional data (Sokal classification, evolutionary stages, and death), treatment data (hematologic remission, cytogenetic remission, molecular remission, and overall survival).

Complete hematologic remission corresponds to the normalization of blood counts with disappearance of clinical signs of disease.

Complete cytogeneticremission corresponds to the absence of the Philadelphia chromosome at the cytogenetic examination (0% Ph + metaphases). It may be partial (1–35% Ph + metaphases) or minor (35%–95% Ph + metaphases) or minimal (96%–100% Ph + metaphases).

The major molecular response corresponds to the disappearance of BCR/ABL transcript.

Patients were seen again for clinical examination, blood count, and biochemical tests once a week for 4 weeks, then every 3 months. Cytogenetic response was assessed every 6 months during the first year, then once a year.

Imatinib mesylate was administered at a dose of 400 mg/day in chronic phase and 600 mg/day in accelerated phase, doses that were adjusted according to tolerance and response. The box of the drug was delivered to the patient in the hospital under medical supervision. The delivery of the second box was preceded by the handing over by the patient of the first empty box and so on. Doses were reduced for neutropenia and thrombocytopenia of grade 3 or 4.

The assessment of the socioeconomic level was done according to indirect criteria which were occupation, number of children, and type of housing.

**Figure 1:**
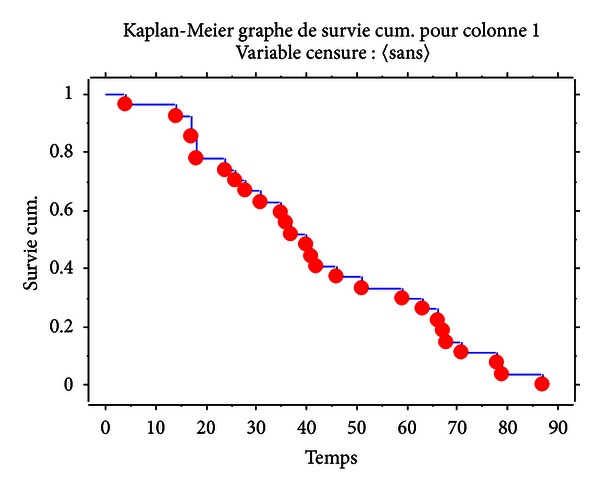
Curve of overall survival in CML patients with additional cytogenetic abnormalities.

Data were analyzed using Epi-Info version 6.04b. The calculation of overall survival was performed according to the Kaplan-Meier method with the existence in the record of a date of inclusion (date of entry) and a point date (date of death or latest news) mentioned in day, month, year (Figure 1). 

## 3. Results 

From May 2005 to October 2011 the diagnosis of CML was made in 91 patients. Among these 91 patients, 27 were carriers of additional cytogenetic abnormalities, that is, 29.7% (Table 2). The epidemiological, clinical, and biological features of those patients are summarized in Table 1. The age ranged from 13 to 68 years with an average of 38 years. The sex ratio was 2.

## 4. Discussion

The mean age of our patients was 38 years with extremes of 13 and 68 years. Our lower average age also reported by other authors [6] could be related to the pyramid of ages of the African people.

The sex ratio was 2. So there were two times more additional cytogenetic abnormalities in men than in women. This male predominance was also noted by Luatti et al. [1] who in his study found 86% of men with an additional cytogenetic abnormality against 59% of men who did not have any with a *P* = 0.02. 

The socioeconomic level of our study population was low or medium. It is this socio-economic class which is taken into account by the GYPAP program, and it is this group that consults in public hospitals, the more affluent preferring private clinics. 

Clinically, the reason for consultation was made by leukocytosis, associated or not with splenomegaly in 100% of cases. 

Generally, our patients were severely symptomatic with a performance status ≥2 in 100%, splenomegaly in 100%, fever in 74%, hepatomegaly in 48%, bone pains in 48% and lymphadenopathy in 22%. Splenomegaly was relatively large, ≥10 cm in 74%, probably due to the long period of consultation. Our patients were characterized by the long period of consultation in clinical hematology because most of them would first consult traditional healers, then a general practitioner. And the time between the date of entry and the date of treatment with imatinib was long for three reasons

- Most of the time patients do not have funds available for biological and radiology assessment; the waiting period before parents accept to help them can often be long.
- Cytogenetics is not performed in Côte d'Ivoire; therefore, the sample has to be sent to France.
- The patient must be enrolled on GIPAP program and must wait till the imatinib is send to him.

All the long delays (consultation and treatment) contribute to alter the condition of the patient, to increase the volume of his spleen, his white blood cell count and the Sokal score, and favor the transition to the accelerate phase of the disease.

These symptoms were mainly found in patients with the accelerated phase of the disease (33%) and/or with a high Sokal score (67%), whereas in the study by Koffi et al. [6] that takes into account all cases of CML diagnosed in our department, there were 59% of performance status quoted at 0 to 1 and 10% who had no splenomegaly.

However, according to Luatti et al. [1], there is no significant difference concerning the size of the spleen in patients with CML with or without additional cytogenetic abnormalities (*P* = 0.10), just as there is no significant difference concerning the Sokal score (*P* = 0.66). 

Biologically, the median of white blood cell count was 314.2 × 10^9^/L, that of platelet count 340 × 10^9^/L; those of polynuclear basophiles, blasts, and promyelocytes were respectively, 5%, 7%, and 8%. Concerning these parameters, according to Luatti et al. [1], there are no significant differences between the two groups of patients with CML with or without additional cytogenetic abnormalities, except for the rate of blasts in the peripheral blood. The median rate of blasts in the peripheral blood was 2.5% versus 1% (*P* = 0.03). 

However, our median rate of white blood cells was higher, and our median hemoglobin rate was lower than the values reported by Braziel et al. [7] The difference in platelets was not significant. 

In our study, we found 29.7% of additional cytogenetic abnormalities. There were 5 cases of variants of Philadelphia chromosome (no. 17 to 21), 5 cases of complex cytogenetic abnormalities (no. 23 to 27), that is, 18.5% each, 4 cases of trisomy 8 (no. 1 to 4), 4 cases of second philadelphia chromosome (22, 24 to 26), that is, 14.8% each and 12 cases of minor cytogenetic abnormalities (no. 5 to 16), that is, 44.4%. Our overall rate of additional cytogenetic abnormalities is relatively high compared to that reported in the literature which is, 5 to 18% [1, 2, 4, 7–10]. For some there was no statistically significant difference between the future and the type of additional cytogenetic abnormalities [2, 11], for others, the difference was significant [4, 12]. In fact, according Fabarius [4], the PFS was 90% versus 50%, and the overall survival to 92% versus 53%, respectively, in those without and with major additional cytogenetic abnormalities with *P* < 0.001. 

The median survival was 40 months. Regarding the survival median that was 40 months, it should be noted that 14 patients had less than 40 months of survival, deaths having occurred precociously due to hematologic toxicity particularly. Three patients were enrolled in 2011.

On the therapeutic level, we obtained 59% of complete hematologic response (CHR) and 41% of partial hematologic response (PHR). Our results are below those of Koffi et al. [6] who had 76% of CHR and 24% of PHR. As for Luatti et al. [1], they achieved 100% of CHR in patients with additional cytogenetic abnormalities. Our low rate of CHR could be related to the Sokal score (33% of intermediate score and 67% of high score). But it should be noted that in the study of Luatti et al. [1], there were 29% of intermediate Sokal score and 38% of high Sokal score in patients with additional cytogenetic abnormalities. 

Concerning the cytogenetic and molecular response, only one patient (3%) achieved a major molecular response. He was carrying a trisomy 8. There was major cytogenetic response (52%), complete cytogenetic response (26%), partial cytogenetic response (26%), minor cytogenetic response (30%), minimal cytogenetic response (15%).

The outcome was fatal in 37%, and the causes of death were dominated by hematologic toxicity (40%), followed by acutisation (30%). In one patient (10%), acutisation occurred at the interruption of the treatment against medical advice. 

Normally, patients during treatment with imatinib are reviewed once a week for 4 weeks, then every 3 months for a complete blood count and biochemical tests. 

Unfortunately, this control rate is not respected by patients, and they return only in case of complications (anemia, neutropenia, thrombocytopenia, fever, etc.). In addition, they often do not have the means to finance a good hematologic resuscitation (transfusions, antibiotics, etc.). This explains the death by hematologic toxicity (Table 3). 

## Figures and Tables

**Table 1:** Epidemiological, clinical, and biological features of patients.

Variables	Numbers (%)
Age (years) mean age 38 years (13–68)	
11–30	13 (48)
31–50	9 (33)
51–70	5 (19)
Sex	
Male	18 (67)
Female	9 (33)
Socioeconomic level	
Low	10 (37)
Average	17 (63)
Reason for consultation	
Hyperleukocytosis	17 (63)
Hyperleukocytosis + splenomegaly	10 (37)
Performance status	
0 and 1	0 (0)
2 and 3	13 (48)
4	14 (52)
Bone pains	
Presence	13 (48)
Fever	
Presence	20 (74)
Hepatomegaly	
Presence	13 (48)
Lymphadenopathy	
Presence	6 (22)
Splenomegaly	
0 cm	0 (0)
1–9 cm	7 (26)
≥10 cm	7 (26)
Hemogram (median values and extremes)	
White Blood Cells (10^9^/L)	341.2 (140–650)
Hemoglobin (g/dL)	8.5 (5.7–12.4)
Platelets (10^9^/L)	340 (57–1992)
Polynuclear basophiles (%)	5 (0–9)
Blasts (%)	7 (0–16)
Promyelocytes (%)	8 (0–18)
Sokal score	
Low	0 (0)
Intermediate	9 (33)
High	18 (67)

**Table 2:** List of additional cytogenetic anomalies at diagnosis of the 27 patients in the study.

No patient	Cytogenetic abnormalities
1	47,XX, +8, t (9;22) (q34;11) [100]
2	47,XY, +8, t (9;22) (q34;11) [1]
3	47,XX, +8, t (9;22) (q34;11) [1]
4	47,XX, +8, t (9;22) (q34;11) [1]
5	46,XY, t (9;22) (q34;q11), add (1) (p36.3) [12]
6	46,XY, t (9;22) (q34;q11), add (2) (p25) [6]
7	46,XX, del (7) (q22;q31), t (9;22) (q34;q11) [100]
8	46,XY, del (7) (q31), t (9;22) (q34;q11) [2]
9	46,XY, del (12) (p12), t (9;22) (q34;q11) [12]
10	46,XY, del (3) (p12p14), t (9;22) (q34;q11) [5]
11	46,XX, del (1) (q12) add (1) (q44), t (9;22) (q34;11) [3]
12	46,XX, t (9;22) (q34;q11), add (13) (p11), add (20) (p13) [23]
13	46,XY, t (9;22) (q34;q11) t (16;20) (q23;q12) [3]
14	46,XX, dup (14) (q22;q32), t (9;22) (q34;q11) [5]
15	46,XX, t (4;11) (q13;p12), t (9;22) (q34;q11) [11]
16	46,XX, inv (12), t (9;22) (q34;11) [100]
17	47,XY, del (13) (q21), t (9;10;22) (q34;p14;q11) + mar 1 [3]
18	46,XY, t (1;3;9;22) (p35;p22;q34;q11) [20]
19	46,XY, t (2;9;22) (q37;q34;q11) [100]
20	46,XY, t (6;9;22) (q22; q34;q11) [3]
21	46,XY, t (3; 9; 22) (q27; q34; q11), der (21) t (21;?) p (12;?) [15]
22	47,XY, der (22), t (9;22) (q34;q11) [100]
23	45-46,XY, t (9;22) (q34;q11), der (12), t(12;14), (q10;q10), add (13), (p11.2), -14, -mar
24	46,XY, t (3; 13) (p25; q14), t(9; 22) (q34; q11) [28]/52, idem, +6,+7,+8,+19,+21, +der(22), t(9;22) (q34;q11)
25	46,XY, del (2) (p22), der (9), t(9;22) (q34;q11), der (22), t(9;22) (q34;q11), +mar 1, +mar 2 [20]
26	46,XY, t (9;22) (q34;q11), der (9), der (22q) [7]/47,XY, idem, der (22) [11]
27	45,XY, -17, t (9;22) (q34;q11), add(19) (q13.4), -mar [19]/45,XY, -17, t (9;22) (q34;q11), add(19) (q13.4) [11]

**Table 3:** Therapeutic and evolutionary features.

Variables	Numbers (%)
Evolutionary phase	
Chronic phase	18 (67)
Accelerated phase	9 (33)
Hematologic remission	
Complete	16 (59)
Partial	11 (41)
Cytogenetic or molecular remission	
Major cytogenetic response (Ph+ ≤ 35%)	14 (52)
Complete cytogenetic response (Ph+ 0%)	7 (26)
Partial cytogenetic response (Ph+ 1%–35%)	7 (26)
Minor cytogenetic response (Ph+ 36%–95%)	8 (30)
Minimal cytogenetic response (Ph+ 96%–100%)	4 (15)
Complete molecular response	1 (3)
Outcome	
Alive	17 (63)
Dead	10 (37)
Causes of death	
Acutisation	3 (30)
Hematologic toxicity	4 (40)
Pulmonary tuberculosis	1 (10)
Epilepsy	1 (10)
Unspecified	1 (10)
Adverse effects grade 3 and 4	
Hematologic toxicity	
Anemia	10 (37)
Leukopenia	9 (33)
Thrombocytopenia	8 (29)
Duration of follow-up (median and extreme values in months)	37 (4–87)
